# Evaluation of non-invasive imaging parameters in coronary microvascular disease: a systematic review

**DOI:** 10.1186/s12880-020-00535-7

**Published:** 2021-01-06

**Authors:** F. Groepenhoff, R. G. M. Klaassen, G. B. Valstar, S. H. Bots, N. C. Onland-Moret, H. M. Den Ruijter, T. Leiner, A. L. M. Eikendal

**Affiliations:** 1 Laboratory of Experimental Cardiology, University Medical Center Utrecht, Utrecht University, Heidelberglaan 100, 3584 CX Utrecht, The Netherlands; 2 Department of Clinical Chemistry and Hematology, University Medical Center Utrecht, Utrecht University, Utrecht, The Netherlands; 3 Department of Epidemiology, Julius Center for Health Sciences and Primary Care, University Medical Center Utrecht, Utrecht University, Utrecht, The Netherlands; 4 Department of Radiology, University Medical Center Utrecht, Utrecht University, Utrecht, The Netherlands

**Keywords:** Coronary microvascular dysfunction, Non-invasive imaging, Reference values, Coronary flow reserve, Myocardial perfusion reserve

## Abstract

**Background:**

Coronary microvascular dysfunction (CMD) is an important underlying cause of angina pectoris. Currently, no diagnostic tool is available to directly visualize the coronary microvasculature. Invasive microvascular reactivity testing is the diagnostic standard for CMD, but several non-invasive imaging techniques are being evaluated. However, evidence on reported non-invasive parameters and cut-off values is limited. Thus, we aimed to provide an overview of reported non-invasive parameters and corresponding cut-off values for CMD.

**Methods:**

Pubmed and EMBASE databases were systematically searched for studies enrolling patients with angina pectoris without obstructed coronary arteries, investigating at least one non-invasive imaging technique to quantify CMD. Methodological quality assessment of included studies was performed using QUADAS-2.

**Results:**

Thirty-seven studies were included. Ten cardiac magnetic resonance studies reported MPRI and nine positron emission tomography (PET) and transthoracic echocardiography (TTE) studies reported CFR. Mean MPRI ranged from 1.47 ± 0.36 to 2.01 ± 0.41 in patients and from 1.50 ± 0.47 to 2.68 ± 0.49 in controls without CMD. Reported mean CFR in PET and TTE ranged from 1.39 ± 0.31 to 2.85 ± 1.35 and 1.69 ± 0.40 to 2.40 ± 0.40 for patients, and 2.68 ± 0.83 to 4.32 ± 1.78 and 2.65 ± 0.65 to 3.31 ± 1.10 for controls, respectively.

**Conclusions:**

This systematic review summarized current evidence on reported parameters and cut-off values to diagnose CMD for various non-invasive imaging modalities. In current clinical practice, CMD is generally diagnosed with a CFR less than 2.0. However, due to heterogeneity in methodology and reporting of outcome measures, outcomes could not be compared and no definite reference values could be provided.

## Background

Patients with angina pectoris (AP) often do not show significant obstructive coronary artery disease (CAD) on coronary angiography (CAG) [1, 2]. Consequently, a cardiac cause of AP complaints is frequently deemed unlikely. Yet, a significant fraction of these patients suffer from cardiac ischemia due to coronary microvascular dysfunction (CMD) [2–6], a condition associated with increased risk of adverse cardiovascular events. This emphasizes the importance of accurate diagnosis of CMD [2, 7–11].

The Coronary Vasomotion Disorders International Study Group (COVADIS) determined the following criteria to diagnose CMD: presence of symptoms and objective documentation of myocardial ischemia, absence of obstructive CAD (< 50% stenosis and/or fractional flow reserve < 0.8) and confirmed reduced coronary flow reserve (CFR) (and/or inducible microvascular spasm). However, assessment of CMD remains challenging, as no tools are available to directly visualize the coronary microvasculature. In fact, the current golden standard to diagnose CMD is invasive measurement of CFR in epicardial arteries without functionally relevant stenosis [12]. The CFR depicts the increase in coronary blood flow in response to vasoactive agents [2, 4, 9] and provides indirect quantification of coronary microvascular blood flow [5, 13].

The invasive nature and high costs of coronary reactivity testing (CRT) initiated the search for a non-invasive alternative to diagnose CMD, including myocardial perfusion reserve index (MPRI) measured using cardiac magnetic resonance imaging (CMR), and CFR using positron emission tomography (PET) and transthoracic echocardiography (TTE) [13–16]. Yet, the cut-off value for CFR to diagnose CMD differs between these modalities, is not well validated and, even though sex-differences in coronary physiology are known, the need for a sex-specific cut-off value remains under debate [17]. To date, a CFR below 2.0 to 2.5 is deemed diagnostic for CMD [9, 16, 18–20].

The (dis)advantages of these non-invasive imaging techniques in the diagnosis of CMD have been discussed extensively before [21]. However, it is unclear which outcome parameters and corresponding cut-off values should be used to diagnose CMD. As such, this systematic review aims to provide an overview of currently reported reference and cut-off values for diagnosing CMD in a non-invasive manner.

## Methods

### Search strategy

On October 15, 2018 the PubMed and EMBASE databases were systematically searched for non-invasive imaging studies on CMD. The search was updated on November 1, 2020. Studies were considered for eligibility without date restriction. The search terms and synonyms of ‘coronary microvascular dysfunction’, ‘nonobstructive coronary disease’ and ‘imaging’, including the imaging modalities CMR, PET and TTE were used. A broad search strategy was performed as studies on CMD are limited and nomenclature of CMD is not standardized. Therefore, search terms were searched for in ‘All Fields’. The detailed search strategy is provided in Additional file 1: Search Strategy.

### Study selection

To assess eligibility, the results from the literature search were initially screened by title and abstract and subsequently for full text. Article selection and data extraction were performed independently by two reviewers (RGMK and FG). Observational studies and randomized controlled trials providing baseline outcome measurements were considered for inclusion.

Studies were included if they enrolled participants with AP (i.e. effort angina or anginal equivalents) and CAG or coronary computed tomography angiography confirmed absent or nonobstructive CAD (based on the definition described in the study protocol of the included studies), or healthy participants without prior history of cardiovascular disease or AP as a control group and reported the results of a non-invasive imaging method with use of pharmacological stress (i.e. flow parameters measured with either CMR, PET or TTE) to diagnose CMD.

Studies written in languages other than English or Dutch, exclusively consisting of participants with comorbidities, i.e. CAD, diabetes mellitus, aortic stenosis or cardiomyopathies, were excluded. Studies were excluded if outcomes were not reported as flow parameters, if patients were stratified according to the outcome of interest or if patient or control groups contained fewer than 10 participants.

### Quality assessment

A methodological quality assessment was performed with the QUADAS-2 (Tool for the Quality Assessment of Diagnostic Accuracy Studies [22]). Studies were assessed for concerns of applicability (‘low’, ‘high’ or ‘unclear’) and for risk of bias (‘low’, ‘high’ or ‘unclear’) on four key domains (patient selection, index test, reference standard and flow and timing). The assessments per domain were combined into an overall risk of bias and concern of applicability.

### Data extraction and analysis

The variables of interest were extracted using a standardized data collection form. Post-hoc evaluations within one clinical trial assessing the same imaging modality were considered as one study. Due to heterogeneity of the included studies, a meta-analysis of the results was not possible.

## Results

### Search results

**Fig. 1:**
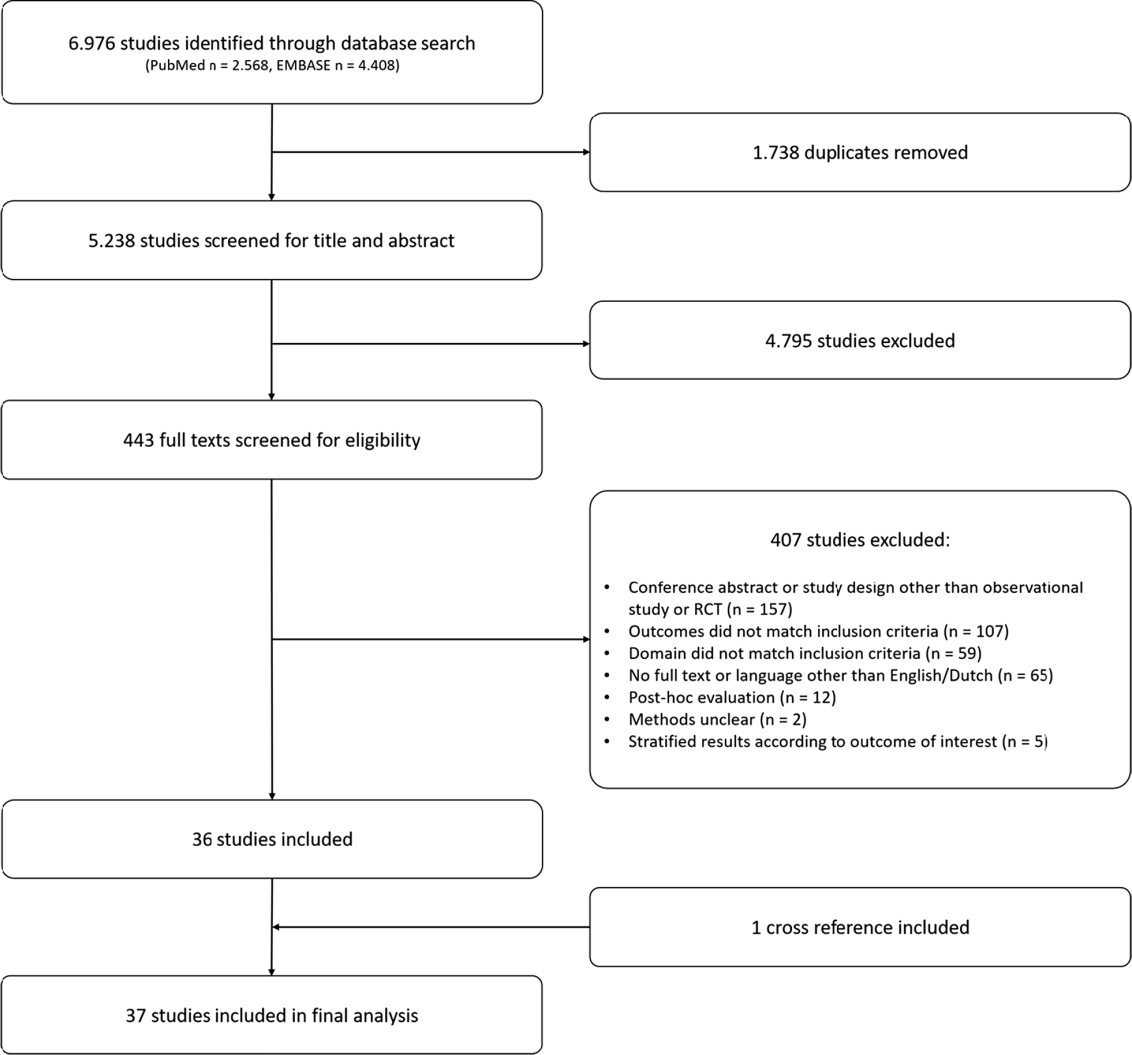
Flow chart study design. *RCT* randomized controlled trial

The search yielded a total of 6976 results, 2568 studies in Pubmed and 4408 studies in Embase. Removal of duplicates resulted in 5238 unique entries. After title and abstract screening, 443 possibly relevant studies were obtained. The full texts of these studies were screened to select those that met the inclusion criteria as provided in the methods section. One relevant study was obtained through cross-reference checking. Thirty-seven studies met the inclusion criteria and were included in the final analysis. The search and inclusion and exclusion of relevant studies are summarized in Fig. 1. The characteristics of the included studies are summarized in Table 1. Quality assessment of included studies showed a clear description of the reference standard was not part of the study protocol in most of the included studies. The full assessment is provided in Additional file 2: Table S1 [22].

**Table 1:** Characteristics of the included studies

Author (year)	Study design	Patient group	N	Mean age (± SD)	Sex (F/M)	Control group	N	Mean age (± SD)	Sex (F/M)	Imaging modality (outcome parameter)	Vasoactive agent used (dose)
Meeder (1997)	Case–control	Patients with syndrome X with typical cardiac chest pain with exercise-induced ischemic-appearing electrocardiographic changes (> 1 mm horizontal ST-T segment depression) and/or reversible myocardial perfusion defects at thallium-201 perfusion scintigraphy and no significant CAD on CAG. Gastro-intestinal causes of chest pain were excluded	25	51 ± 9	16/9	Healthy volunteers	21	42 ± 13	8/13	PET (MPR), N-13 ammonia	Dipyridamole (0.56 mg/kg per 4 min)
Bottcher (1999)	Case–control	Angina pectoris and positive stress ECG, normal CAG without risk factors for CAD	25	53 ± 7	25/0	Healthy age and sex matched volunteers	15	54 ± 10	15/0	PET (CFR), N-13 ammonia	Dipyridamole (0.56 mg/kg per 4 min)
Buus (1999)	Case–control	Typical effort angina, positive stress ECG, normal CAG and TTE. No history of hypertension or diabetes mellitus	16	56.6 ± 1.2	13/3	Healthy subjects (recruited among blood donors and hospital staff)	15	53.5 ± 1.1	12/3	PET (CFR), N-13 ammonia	Dipyridamole (0.56 mg/kg per 4 min)
Panting (2002)	Case–control	Typical effort angina, abnormal stress ECG, normal CAG recruited from Women’s Heart Disease Clinic at Royal Brompton Hospital (London)	20	55.9 ± 10.5	16/4	Healthy age and sex matched subjects, no history of chest pain and low cardiovascular risk profile. No SPECT or CAG was performed	10	57.9 ± 7.4	8/2	CMR (MPRI), 1.5 T	Adenosine (140 mcg/kg/min for 6 min)
Marroquin (2003)	Case–control	Women with chest pain and epicardial coronaries that were angiographically normal or with only minimal luminal irregularities (< 50% stenoses) who were enrolled in the WISE study at the University of Pittsburgh	34	52.1 ± 10.0	34/0	Healthy age-matched women	9	50.4 ± 12.2	9/0	PET (CFR), 13-N ammonia	Adenosine (140 mcg/kg/min for 4 min)
De Vries (2006)	Case–control	Typical chest pain and normal CAG. Exclusion: LBBB on ECG, first degree AV block and diabetes mellitus	42	58 ± 12	26/16	Healthy volunteers without chest pain or CAD	21	N/A	N/A	PET (CFR), N-13 ammonia	Dipyridamole (0.56 mg/kg per 6 min)
Graf (2006)	Case–control	Typical angina, normal CAG and positive stress ECG or SPECT, exclusion of myocardial or valvular disease by TTE. Exclusion: diabetes mellitus	58	58 ± 10	39/19	N/A	N/A	N/A	N/A	PET (CFR), N-13 ammonia	Dipyridamole (0.56 mg/kg per 4 min)
Pärkkä (2006)	Cross-sectional/descriptive	N/A	N/A	N/A	N/A	Male volunteers, nonsmoking. One patient with hypertension, others no history of cardiovascular disease	18	40.0 ± 14.4	0/18	CMR (MPR), 1.5 T PET (MPR), 15O-labeled water	Dipyridamole (0.56 mg/kg per 4 min)
Wöhrle (2006)	Case series	Typical angina pectoris and normal CAG	12	61.8 ± 8.2	7/5	N/A	N/A	N/A	N/A	CMR (MPRI), 1.5 T	Adenosine (140 mcg/min/kg for 3 min)
Galiuto (2007)	Case–control	Typical effort angina, positive stress ECG and normal CAG. Exclusion: moderate to severe hypertension, diabetes mellitus, other heart disease or contraindications to adenosine infusion	17	55 ± 10	9/8	Healthy subjects age and sex matched. Exclusion: moderate to severe hypertension, diabetes mellitus, other heart disease or contraindications to adenosine infusion	17	55 ± 10	10/7	TTE (CFR), distal LAD with pulse-wave Doppler	Adenosine (140 mcg/kg in 90 s)
Graf (2007)	Case–control	Typical angina, normal CAG and positive stress ECG or SPECT. Myocardial or valve disease excluded by TTE. Exclusion: diabetes mellitus and other major diseases	79	58 ± 10	52/27	Atypical chest pain, normal CAG and negative stress test. Myocardial or valve disease excluded by TTE. Exclusion: diabetes mellitus and other major diseases	10	53 ± 11	6/4	PET (CFR), N-13 ammonia	Dipyridamole (0.56 mg/kg per 4 min)
Vermeltfoort (2007)	Case series	Effort angina, positive stress ECG or SPECT and normal CAG. Exclusion: history of heart disease, hypertension, diabetes mellitus, absence of pain without medication, contra-indication for CMR	20	55 ± 11	15/5	N/A	N/A	N/A	N/A	CMR (MPRI), 1.5 T	Adenosine (140 mcg/kg/min for 3 min)
Cemin (2008)	Case–control	N/A	N/A	N/A	N/A	Healthy volunteers with low pretest likelihood of coronary disease who were undergoing CAG	14	62.6 ± 9.1	8/6	TTE (CFR), distal LAD with pulse-wave Doppler	Adenosine (140 mcg/kg/min for 5 min)
Lanza (2008)	Case–control	Effort angina, positive stress test and normal CAG. Exclusion: history of heart disease or systemic diseases	18	58 ± 7	11/7	Healthy volunteers, enrolled from the non-medical hospital staff, comparable in age and sex	10	54 ± 8	6/4	TTE (CFR), mid-distal LAD with Doppler spectral tracing	Adenosine (140 mcg/kg/min for 90 s)
Di Monaco (2009)	Case–control	Patients presenting with effort angina, positive stress test and normal CAG in a university hospital. Exclusion: previous enrollment in SPECT study	29	59 ± 7	18/11	Healthy subjects, age and sex matched	20	56 ± 6	12/8	TTE (CFR), mid-distal LAD with Doppler spectral tracing	Adenosine (140 mcg/kg/min for 90 s)
Mehta (2011)	RCT	Women with chest pain and abnormal stress testing, no obstructive CAD (< 50%) on CAG. Exclusion: renal failure or hepatic insufficiency, contraindication to withholding nitrates, calcium channel agents and beta-adrenergic blockers for 24 h, contraindication to CMR and use of drugs inhibiting CYP3A	20	57 ± 11	20/0	N/A	N/A	N/A	N/A	CMR (MPRI), 1.5 T	Adenosine (140 mcg/kg/min for 5 min)
Scholtens (2011)	Case–control	Patients submitted for PET analysis because of typical chest pain, positive stress ECG and normal CAG	14	55 (34–76) *Median (range)*	10/4	Healthy subjects	13	58 (48–73) *Median (range)*	11/2	PET (MPR), N-13 ammonia	Adenosine (140 mcg/kg/min for 6 min)
Sestito (2011)	Case–control	Patients with a history of effort angina, positive stress test and normal CAG undergoing clinical follow-up. Exclusion: other cardiac or systemic disease	71	56 ± 9	48/23	Healthy volunteers enrolled from the nonmedical hospital staff, age and sex matched	20	52 ± 7	11/9	TTE (CBF), mid-distal LAD with Doppler spectral tracing	Adenosine (140 mcg/kg/min for 90 s)
Vaccarino (2011)	Cohort	N/A	N/A	N/A	N/A	Middle aged male-male twin pairs from the Vietnam Era Twin Registry without previous history of CAD	268	54.0 (53.5–54.6) *Median (range)*	0/268	PET (CFR), N-13 ammonia	Adenosine (140 mcg/kg/min for 4 min)
Vermeltfoort (2011)	Case series	N/A	N/A	N/A	N/A	Healthy subjects without cardiovascular risk factors	27	41 ± 13	16/11	PET (CFR), 15O- labeled water	Adenosine (140 mcg/kg/min for 3 min)
Di Franco (2012)	Case–control	Effort angina, positive stress test and normal CAG enrolled at outpatient ambulatory clinic	14	61 ± 5	9/5	Healthy subjects enrolled from patients referred to outpatient cardiology clinic for palpitations or evaluation of cardiovascular risk, age and sex matched	14	61 ± 3	7/7	TTE (CBF), mid-distal LAD with Doppler spectral tracing	Adenosine (140 mcg/kg/min for 90 s)
Karamitsos (2012)	Case–control	Typical effort angina, abnormal stress ECG and normal CAG. Exclusion: diabetes mellitus, hypertension and other cardiac or systemic disease	18	62 ± 8	15/3	Healthy individuals without cardiovascular risk factors	14	58 ± 6	11/3	CMR (CFR), 3 T	Adenosine (140 mcg/kg/min for 4–5 min)
Uusitalo (2013)	Cohort	N/A	N/A	N/A	N/A	Healthy men ≤ 45 years from healthy control groups of two earlies reported studies. Exclusion: hypertension, smoking, diabetes mellitus, obesity or history of atherosclerotic disease	77	35.3 ± 3.9	0/77	PET (CFR), 15O-labeled water	Adenosine (dose not reported) or dipyridamole (0.56 mg/kg per 4 min)
Nelson (2014)	Case–control	N/A	N/A	N/A	N/A	Healthy age matched women with no cardiac risk factors	15	56 (SD not available)	15/0	CMR (MPRI), 1.5 T	Adenosine (140 mcg/kg 3–4 min)
Thomson (2015)	Case–control	Women with signs and symptoms of ischemia with clinically indicated CRT; part of NHLBI-sponsored WISE-Coronary Vascular Dysfunction study performed at Cedars-Sinai Medical Center or the University of Florida. Exclusion: history of obstructive CAD (> 50% stenosis) or other cardiac disease, contraindications to CMR	118	53.9 ± 11.4	118/0	Healthy age matched women with no cardiac risk factors	21	53.6 ± 9.1	21/0	CMR (MPRI), 1.5 T	Adenosine (140 mcg/kg from 2 min prior until completion of first pass perfusion imaging)
Tagliamonte (2015)	RCT	Signs and symptoms of myocardial ischemia, no CAD (< 70% stenosis on CAG). Myocardial ischemia confirmed by SPECT, assigned to placebo. Exclusion: renal failure or hepatic insufficiency, LBBB on ECG, use of drugs inhibiting CYP3A, other cardiac disease As above, assigned to ranolazine	29 29	65 ± 11 66 ± 10	9/20 10/19	N/A	N/A	N/A	N/A	TTE (CFR), distal LAD with Doppler spectral tracing	Dipyridamole (up to 0.84 mg over 6 min)
Wu (2015)	RCT	Diagnosis of CMD based on the presence of typical effort angina, exercise-induced ST segment depression (> 1 mm), normal CAG, absence of any specific cardiac disease including vasospastic angina and reduced CFR (< 2.0) measured by TTE with adenosine	20	60 ± 8	17/3	N/A	N/A	N/A	N/A	TTE (CBFVR), mid-distal LAD with Doppler spectral tracing	Nitroglycerin (25 mcg)
Bairey Merz (2016)	RCT	Symptoms due to ischemia objectified by stress testing, no obstructive CAD (< 50% stenosis on CAG) with abnormal CRT (CFR < 2.5) or CMR (MPRI < 2.0). Exclusion: other cardiac disease or life expectancy < 4 years, contraindication for CMR or use of CYP3A4 inhibitors	128	55.2 ± 9.8	123/5	N/A	N/A	N/A	N/A	CMR (MPRI), 1.5 T	Adenosine (not reported)
Bakir (2016)	Case series	N/A	N/A	N/A	N/A	Women without signs and symptoms of myocardial ischemia and absence of cardiovascular risk factors recruited at Cedars-Sinai Medical Center based on their age and hormone-use status to match CMD subjects in the WISE trial. Exclusion: contraindication to CMR or adenosine, renal disease	20	54 ± 9	20/0	CMR (MPRI), 1.5 T	Adenosine (140 mcg/kg/min for 3–4 min)
Mygind (2016)	Case series	Women referred for clinically indicated CAG due to angina-like chest pain form the Patient Analysis & Tracking System in eastern Denmark. Inclusion: CAD < 50% stenosis. Exclusion: other cause of chest pain more likely, no cardiac disease, life-expectancy < 1 year	963	62.1 ± 9.7	963/0	N/A	N/A	N/A	N/A	TTE (CFVR), LAD with pulsed-wave Doppler Contrast (SonoVue) used in case of difficulty visualizing LAD	Dipyridamole (0.84 mg/kg in 6 min)
Anchisi (2017)	Case series	Recurrent chest pain, ECG alterations at ergometry and normal CAG. Exclusion: other cardiac disease and previous revascularization. Setting: Cardiology Unit of Azienda Ospedaliera-Universitaria ‘Maggiore della Carità’ in Novara	16	64 ± 11	10/6	N/A	N/A	N/A	N/A	TTE (CFR), color Doppler flow mapping, mid-distal LAD	Dipyridamole (0.84 mg/kg per 6 min)
Jaarsma (2017)	Case–control	Typical effort angina, positive stress ECG and normal CAG (stenosis < 25%), consecutively enrolled at Maastricht University Medical Center. Exclusion: contraindications for CMR or adenosine. One patient excluded due to poor image quality	13	65 ± 9	7/6	N/A	N/A	N/A	N/A	CMR (MPR), 3 T	Adenosine (140 mcg/kg/min for 4 min)
Michelsen (2017)	Case–control	Women with angina-like chest pain and no significant obstructive CAD (< 50% stenosis) and with successful TTE examination, randomly selected from the iPOWER study cohort	95 102	61.8 ± 8.8 (in all 107 particpants)	95/0 102/0	N/A	N/A	N/A	N/A	PET (MBFR), Rubidium-82; TTE (CFVR), LAD with pulse-waved Doppler; Contrast (SonoVue) used in case of difficulty visualizing LAD	Adenosine (0.84 mg/kg per 6 min) Dipyridamole (0.84 mg/kg per 6 min)
Liu (2018)	Case–control	Patients with angina and suspected or known CAD referred for outpatient diagnostic CAG without obstructive CAD on CAG	22	65 ± 8	8/14	Healthy age-matched subjects	20	61 ± 7	7/13	CMR (MPRI), 1.5 T or 3 T	Adenosine (140 mcg/kg/min for ≥ 3 to 6 min)
Liu (2018)	Case–control	Patients with stable angina and suspected CAD referred for outpatient diagnostic CAG in a tertiary referral hospital with FFR ≥ 0.8 and IMR ≥ 25 U	13 11	N/A N/A	N/A N/A	Healthy volunteers	30	51 ± 15	9/21	CMR, 1.5 or 3 T	Adenosine (140 mcg/kg/min, for ≥ 3 to 6 min)
Zorach (2018)	Case–control	Patients with typical effort angina and no CAD (< 50% stenosis) on CAG and with risk factors for CMD (diabetes mellitus or metabolic syndrome) recruited from the University of Virginia Health System	46	57.5 ± 11.2	34/12	Healthy controls without risk factors for CMD	20	53.4 ± 11.9	12/8	CMR (MPR), 1.5 T	Regadenoson
Rahman (2019)	Case–control	Patients undergoing elective diagnostic angiography for investigation of exertional chest pain and nonobstructive coronary artery disease (< 30% diameter stenosis and/or fractional flow reserve > 0.80) with CFR < 2.5	38	2.01 ± 0.41	N/A	Patients undergoing elective diagnostic angiography for investigation of exertional chest pain and nonobstructive coronary artery disease (< 30% diameter stenosis and/or fractional flow reserve > 0.80) with CFR > 2.5	27	2.68 ± 0.49	N/A	CMR (MPR), 3 T	Adenosine (140 mcg/kg/min for 3 min)

*CABG* coronary artery bypass grafting, *CAD* coronary artery disease, *CAG* coronary angiography, *CRT* coronary reactivity testing, *ECG* electrocardiogram, *F* female, *FFR* fractional flow reserve, *IMR* index of microcirculatory resistance, *ISMN* isosorbide-5-mononitrate, *LAD* left anterior descending coronary artery, *LBBB* left bundle branch block, *M* male, *NHLBI-sponsored WISE* National Heart, Lung, and Blood Institute sponsored women’s ischemia syndrome evaluation, *RCT* randomized controlled trial

### Demographic information

The number of patients included in each study was generally small, with a median study population of 22 patients (range 11 to 963, 89% women) and median of 18 controls (range 10 to 268, 33% women). The mean age in patient groups ranged from 50.0 ± 7.0 to 66.0 ± 10.0 years of age and 35.3 ± 3.9 to 62.6 ± 9.1 years of age in control groups. The specific demographic information per study is summarized in Table 1.

### Flow parameters

Different flow parameters were reported (Table 2). In CMR studies, the myocardial perfusion reserve index (MPRI) was most often reported. Other parameters reported were myocardial perfusion reserve (MPR) and CFR. MPR is defined as the ratio between the relative upslope of myocardial signal intensity (obtained with the use of gadolinium as contrast agent) during stress and rest. In contrast to MPR, the MPRI is corrected for left ventricular contrast signal intensity, allowing for a reduction in signal differences within the image and intra-individual level differences in signal intensity due to heart rate and blood pressure [6, 23, 24]. As such, MPRI is often the preferred outcome measure as it seems to be more accurate in quantifying coronary microvascular blood flow. In one study, CMR-derived CFR results were presented [25]. CFR was calculated and measured in the exact same way as the MPR and can therefore be considered as a synonym of MPR.

**Table 2:** Overview of outcome parameters considered in this systematic review

Imaging method	Parameters	Definition
Cardiac magnetic resonance imaging (CMR)	Myocardial perfusion reserve index (MPRI)	MBF_stress_/MBF_rest_ * LV contrast signal intensity
	Myocardial perfusion reserve (MPR)	MBF_stress_/MBF_rest_
	Coronary flow reserve (corrected for rate pressure product) (CFR (corrected for RPP))	MBF_stress_/MBF_rest_ * (HR * SBP/10.000)
Positron emmission tomography (PET)	Coronary flow reserve (corrected for rate pressure product) (CFR (corrected for RPP))	MBF_stress_/MBF_rest_ * (HR * SBP/10.000)
	Myocardial perfusion reserve (MPR)	MBF_stress_/MBF_rest_
	Myocardial flow reserve (MFR)	MBF_stress_/MBF_rest_
Transthoracic echocardiography (TTE)	Coronary flow reserve (CFR)	MBF_stress_/MBF_rest_
	Coronary flow velocity reserve (CFVR)	MBF_stress_/MBF_rest_
	Coronary blood flow (CBF)	MBF_stress_/MBF_rest_

*HR* heart rate, *LV* left ventricular, *MBF*_*rest*_ myocardial blood flow in resting conditions, *MBF*_*stress*_ myocardial blood flow during hyperemic circumstances, *SBP* systolic blood pressure

In PET studies microvascular function was usually quantified with CFR. Other outcome parameters were MPR or myocardial flow reserve (MFR). MPR and MFR were calculated based on the same methods and measurements as CFR and could therefore be used interchangeably. CFR was defined as the ratio between hyperemic and resting myocardial blood flow (MBF) [26, 27] which was expressed in ml/min/g [28]. CFR was often corrected for rate pressure product (RPP), defined as heart rate multiplied by systolic blood pressure and represents cardiac metabolic demand. This correction is recommended as it reduces variability in outcomes due to person-level differences in systolic blood pressure and heart rate [2, 27].

In TTE studies CFR was used. Similar to PET and CMR, a variety of equivalent terms were reported, namely CFR, coronary blood flow (CBF) and coronary flow velocity reserve (CFVR). CFR, CBF and CFVR were all defined as the ratio of peak stress and rest coronary blood flow velocities (CBFV), usually obtained by spectral Doppler measurements.

### CMR imaging

**Fig. 2:**
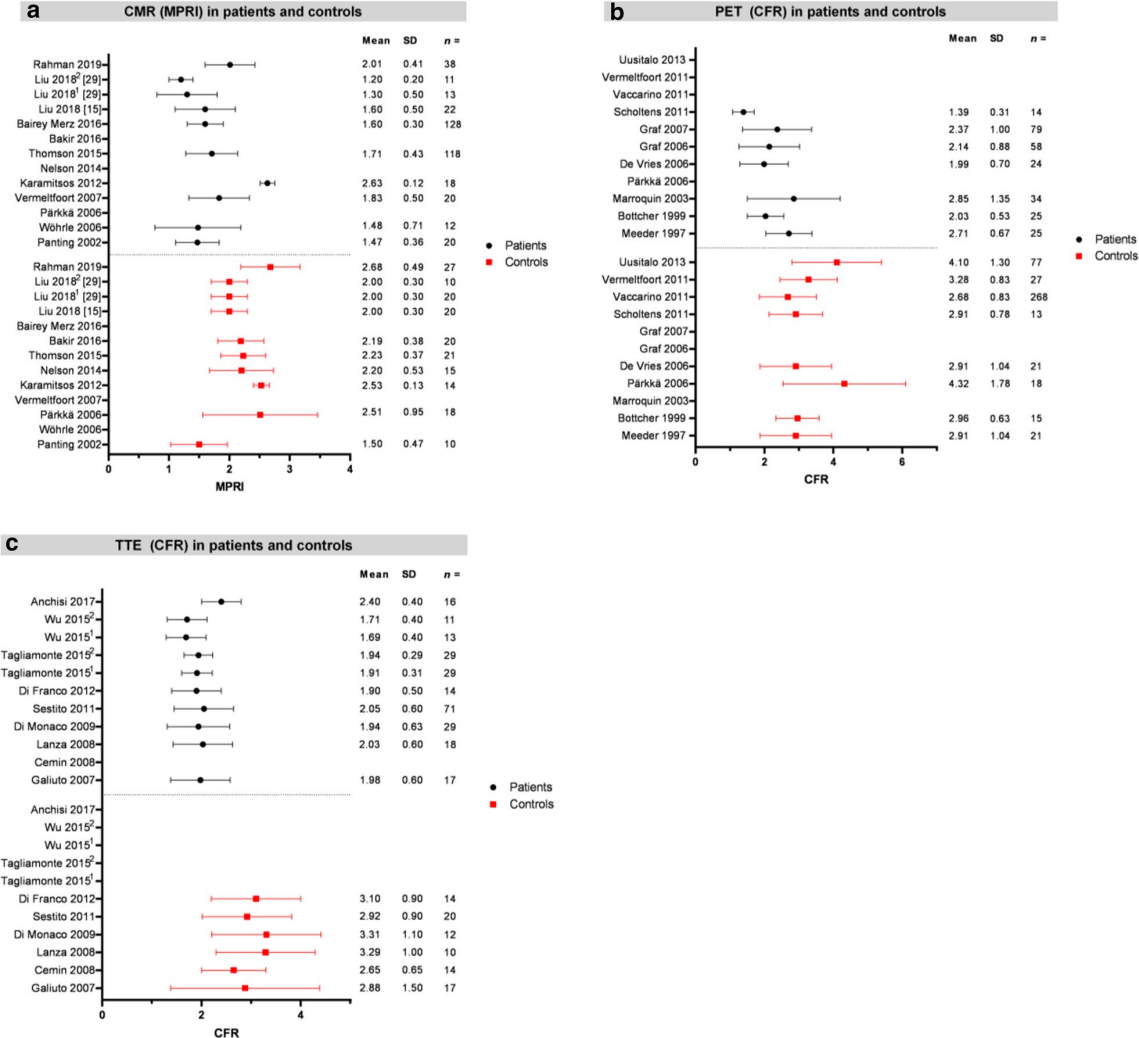
Overview of study outcomes presented as mean ± SD in patients and controls for MPRI by CMR (**a**), CFR by PET (**b**) and CFR by TTE (**c**). For CMR. Error bars are not shown for some studies as some only assessed patient or control subjects. Studies with multiple patient or control groups are indicated by numbers. *CFR* coronary flow reserve, *CMR* cardiac magnetic resonance imaging, *MPRI* myocardial perfusion reserve index, *PET* positron emission tomography, *TTE* transthoracic echocardiography

CMR was used to diagnose CMD in 15 of the 37 included studies (Additional file 3: Table S2) [6, 15, 23, 25, 29–39]. CMR results are mostly expressed as the MPRI (*n* = 11). The other outcome parameters mentioned were MPR and CFR (*n* = 5). One study assessed MPRI as well as MPR [29]. Patient groups were globally comparable as all studies included patients with AP without CAD on CAG. Absolute mean transmural mean MPRI values in patient groups ranged from 1.47 ± 0.36 to 2.01 ± 0.41. In controls, mean MPRI ranged from 1.50 ± 0.47 to 2.68 ± 0.49. The results of CMR studies with MPRI as outcome parameter in patients and controls are summarized in Fig. 2a.

### PET imaging

A total of 13 studies used PET to quantify coronary microvascular function (Additional file 4: Table S3) [16, 35, 40–50]. PET studies reporting mean CFR as outcome measure, mean CFR ranged from 1.39 ± 0.31 to 2.85 ± 1.35 in patient groups. In the control group, mean CFR ranged from 2.68 ± 0.83 to 4.32 ± 1.78. The results of PET studies with CFR as outcome parameter in patients and controls are summarized in Fig. 2b.

### TTE imaging

In 11 studies CMD was assessed by TTE (Additional file 5: Table S4) [16, 51–60]. All studies calculated CFR as the ratio of basal and hyperemic diastolic flow velocity measured in the left anterior descending coronary artery (LAD). In the included TTE studies, patient groups were comparable with regard to inclusion of patients with AP and no or nonobstructive CAD on CAG (Table 1). Two RCTs were included, mentioning CFR at baseline. A mean CFR of 1.69 ± 0.40 to 2.40 ± 0.40 was found in patients with angina and no CAD on CAG, whereas healthy control subjects show a higher mean CFR of 2.65 ± 0.65 to 3.31 ± 1.10. An overview of the CFR outcomes of TTE studies in patients and controls is presented in Fig. 2c.

### Sex differences

Only one of the included studies compared outcomes between men and women. Sestito et al. [56] determined CBF (defined as the ratio of diastolic CBF velocity at peak stress and rest) using TTE in 71 patients diagnosed with CMD (48 women, 67.6%). No significant difference in CBF was found between men and women (CBF 2.09 ± 0.60 and 2.03 ± 0.50, respectively). Furthermore, the proportion of women as compared to men in the patient groups was much larger (89%) as compared with the control population (33%).

## Discussion

We provided an overview of currently used non-invasive imaging techniques and corresponding reference values for CMD in patients with AP and no or nonobstructive CAD as well as healthy subjects. We found quite some differences between reported non-invasive imaging parameters to assess CMD, which we have summarized in Fig. 2. These differences reflect the large heterogeneity between the studied population as well as the rapidly developing imaging techniques and protocols per imaging technique, which result in a variety of different study protocols. Due to the heterogeneity between the included studies we were unable to perform a formal meta-analysis and provide clear clinically applicable cut-off values to diagnose CMD.

MPRI was found to correlate well with invasive measurements obtained with CRT, such as index of microcirculatory resistance and CFR [15, 23]. Therefore, MPRI could potentially serve as a non-invasive alternative to CRT. Current literature proposes two different cut-off values, namely 1.40 and 1.84 [6, 15], corresponding with the results found in this review. However, the results of this review suggest a grey area of MPRI values, as some overlap is seen between MPRI in patients and healthy controls. Stress MBF values can aid in differentiating CMD from normal coronary microvascular function. Liu et al. [15] have shown that a decreased stress MBF (i.e. less than 2.30 ml/min/g) is suggestive of CMD in patients with inconclusive MPRI values. Furthermore, some CMR studies now investigate the clinical applicability of quantitative myocardial tissue characterization with rest and stress T1 mapping as an alternative [29, 33]. Ischemic myocardial tissue can be differentiated from healthy tissue based on distinct properties at T1 mapping during rest and stress conditions, without the use of contrast agents. However, the diagnostic value of T1 mapping in diagnosing CMD still needs extensive validation [29].

Currently, PET is the most frequently applied and validated non-invasive imaging technique in quantifying microvascular blood flow. PET is considered the golden standard of non-invasive diagnosis of CMD, although discordance between invasive fractional flow reserve (FFR) and non-invasive CFR is reported in up to 30% of cases [16, 26, 27, 61]. CMD is generally diagnosed with a CFR less than 2.0 if corrected for RPP or less than 2.5 if uncorrected [28, 35, 45, 47]. However, no evidence-based cut-off values for CFR in PET are available yet. Similarly, no cut-off values for CFR in TTE have yet been determined and generally a cut-off value of less than 2.0 for the diagnosis of CMD is applied [18, 58, 62–65]. The study of Hildick-Smith et al. showed CFR with use of adenosine stress TTE to be well above this applied cut-off value, i.e. a mean CFR 3.7 in healthy controls and 5.9 in athletes [66]. However these study population comprised men of 27 years of age and could therefore not be directly used as reference value for the, mainly older and female, population of interest at risk for CMD. TTE assessment of CFR with Doppler echocardiography has been validated against intracoronary Doppler measurements and outcomes correlate well [2, 5, 60, 67].

### Causes of heterogeneity in measured outcome parameters

#### Patient groups

The heterogeneity in outcomes observed in this systematic review is most likely the result of differences in inclusion criteria applied across several studies and differences in the use of imaging techniques. Although most studies investigated patients with typical AP and no or nonobstructive CAD during diagnostic CAG, the setting in which participants were recruited was not reported clearly. Furthermore, the definition of no or nonobstructive CAD and the control population was often unclear and, if documented, heterogeneous among the included studies (Additional file 6: Table S5). Therefore, a more homogeneous definition could not be applied in the search method. Hence, we suggest the use and documentation of standardized criteria as reported by COVADIS [12].

#### Methodological differences

Unclarity of the used reference standard, as reflected by the risk of bias assessment (Additional file 2: Table S1), may have introduced significant bias. Moreover, it was often unclear whether researchers were blinded to the reference standard when interpreting results from the index test.

Furthermore, measurement of MPRI in CMR might contribute to the inconsistent results observed in this systematic review. MPRI can be measured transmural, but also subendo- or epicardial. Several studies show subendocardial MPRI to be decreased more often than epicardial MPRI in CMD patients [34, 39, 64, 68], which might indicate subendocardial MPRI to be more valuable in diagnosing CMD as compared with epicardial or transmural MPRI. Unfortunately, in this systematic review only transmural MPRI values were included.

Regarding PET, correction for RPP is not standard which results in decreased comparability of outcomes. Moreover, the use of different radioactive tracers (15O-water, 13N-ammonia and Rubidium-82) could result in varying outcomes due to differences in characteristics and processing of images [14, 27, 28]. The use of a specific radiotracer might require a specific cut-off value to diagnose CMD [14]. Similar concerns apply to the use of various vasoactive agents to achieve hyperemia in stress perfusion imaging. Adenosine and dipyridamole are most commonly administered to achieve hyperemia. However, adenosine seems to be superior to dipyridamole with regard to attaining maximal hyperemia and their effects are not identical. Therefore the use of the different types and doses of vasoactive agent could have contributed to the differences found in the outcome parameters [27, 69, 70].

Lastly, this systematic review highlights the discordance in nomenclature and reporting of outcomes. Standardization of outcome parameters reported could increase comparability of studies assessing reference values for the diagnosis of CMD.

#### Sex differences

In the present analysis women were highly represented in the patient groups (89%) compared to control groups (33%). Therefore, sex differences could contribute to discrepancies between studies resulting in decreased comparability between CFR and MPRI measurements in patient and control groups. Kobayashi et al. [17] measured coronary vascular diameter with quantitative CAG and intravascular ultrasound in patients with AP and nonobstructive CAD and found a smaller vascular diameter in women. Furthermore, they showed a significantly higher resting CBF in women. The latter is consistent with findings by Opstal et al. [71] and Chareonthaitawee et al. [72] who studied coronary blood flow in healthy subjects with 13N-ammonia PET (*n* = 206) and 15O-water PET (*n* = 169), respectively. These findings suggest sex differences in flow parameters. High resting myocardial flow volumes could decrease CFR (in PET and TTE) or MPRI (in CMR) in women compared to men as flow parameters are determined as the ratio of stress and rest perfusion. Although sex differences in resting MBF and CFR have been observed in invasive CRT [3, 17, 73], only one of the included studies included assessed sex differences regarding CBF and reported no significant sex differences [56]. These findings are consistent with another study comparing non-invasive CFR between men and women using PET [74]. Therefore, further research is needed to establish whether or not sex-specific cut-off values are required for the non-invasive diagnosis of CMD.

### Recommendations for future research

The studies included in this review show heterogeneity in study methodology and outcome. This contributes to the discrepancies in outcomes between studies and to the lack of consensus regarding definition and cut-off values for CMD in non-invasive imaging modalities. We emphasize the need for large validation studies and suggest standardization of outcome parameters to reduce heterogeneity and increase comparability of studies. This is needed to provide clinically applicable, possibly sex-specific, reference values for the diagnosis of CMD in the future.

Furthermore, during this systematic review, we found several other imaging modalities that are studied for their potential to diagnose CMD, such as myocardial contrast echocardiography (MCE) [63, 75, 76], CT-perfusion [77] and absolute quantification of myocardial perfusion by CMR [78]. Even though, current evidence is still limited so the clinical significance and applicability in regular care of these modalities in CMD diagnosis remains unclear, current research shows promising results. For example, Bechsgaard et al. [77] studied CT myocardial perfusion in women with angina and no obstructive CAD, (defined as < 50% stenosis), in comparison with female controls. They showed CT-perfusion is able to identify decreased global myocardial perfusion and impaired increase of myocardial blood flow during adenosine provocation in women with angina and non obstructive CAD as compared with the control group. The use of CT-perfusion in addition to the commonly performed CCTA could in the future play an important role in the evaluation of CMD early in the evaluation of patients with angina.

### Study limitations

The number of studies investigating non-invasive imaging techniques to diagnose CMD is limited. As such, the results of this systematic review are based on limited data. Hence, only an indication of reference and cut-off values could be provided. Furthermore, a formal meta-analysis could not be performed due to heterogeneity of included studies. In addition, the risk of selection bias in the included studies was high. Also, the heterogeneity in the definition of non obstructive CAD might also have impacted the results as the included studies comprise patients with both completely normal or non obstructed coronaries. Unfortunately, as the definition of nonobstructive CAD was unclear or heterogeneous, it was not possible to separately analyse outcomes for patients with normal coronaries as compared to nonobstructive CAD. These analyses would have been of additive value as in patients with nonobstructive CAD there might still be epicardial stenosis that could impact myocardial blood flow. These limitations emphasize the importance of standardization of imaging protocols and analyses, patient selection and reporting of outcome measurements to obtain reliable and clinically relevant cut-off values for CMD.

## Conclusions

This systematic review provided an overview of currently used parameters and cut-off values for CMD in patients with AP and no or nonobstructive CAD as well as healthy subjects. However, no definite cut-off values could be determined as no meta-analysis could be performed due to heterogeneity of studies investigating non-invasive imaging techniques in CMD.

## Supplementary Information

**Additional file 1.** Search Strategy.

**Additional file 2: Table S1.** Table S2 Risk of bias assessment according to QUADAS-2.

**Additional file 3: Table S2.** Quantification of coronary microvascular dysfunction in CMR studies.

**Additional file 4: Table S3.** Quantification of coronary microvascular dysfunction in PET studies. Correction for RPP is indicated by + or –.

**Additional file 5: Table S4.** Quantification of coronary microvascular dysfunction in TTE studies.

**Additional file 6: Table S5.** Definition of nonobstructive CAD in included studies.

## Data Availability

The datasets used and/or analysed during the current study are available from the corresponding author on reasonable request.

## Abbreviations

AP: Angina pectoris
CAD: Coronary artery disease
CAG: Coronary aniography
CFR: Coronary flow reserve
CMD: Coronary microvascular dysfunction
CMR: Cardiac magnetic resonance imaging
COVADIS: Coronary Vasomotor Disorders International Study Group
CRT: Coronary reactivity testing
MBF: Myocardial blood flow
MPR: Myocardial perfusion reserve
MPRI: Myocardial perfusion reserve index
PET: Positron emission tomography
TTE: Transthoracic echocardiography
