# Correction to: Evaluation of non-invasive imaging parameters in coronary microvascular disease: a systematic review

**DOI:** 10.1186/s12880-021-00686-1

**Published:** 2021-10-20

**Authors:** F. Groepenhof, R. G. M. Klaassen, G. B. Valstar, S. H. Bots, N. C. Onland-Moret, H. M. Den Ruijter, T. Leiner, A. L. M. Eikendal

**Affiliations:** 1 Laboratory of Experimental Cardiology, University Medical Center Utrecht, Utrecht University, Heidelberglaan 100, 3584 CX Utrecht, The Netherlands; 2 Department of Clinical Chemistry and Hematology, University Medical Center Utrecht, Utrecht University, Utrecht, The Netherlands; 3 Department of Epidemiology, Julius Center for Health Sciences and Primary Care, University Medical Center Utrecht, Utrecht University, Utrecht, The Netherlands; 4 Department of Radiology, University Medical Center Utrecht, Utrecht University, Utrecht, The Netherlands

## Correction to: BMC Med Imaging (2021) 21:5 10.1186/s12880-020-00535-7

Following the publication of the original article [1] the authors became aware of an error in Fig. 2.

**Fig. 2:**
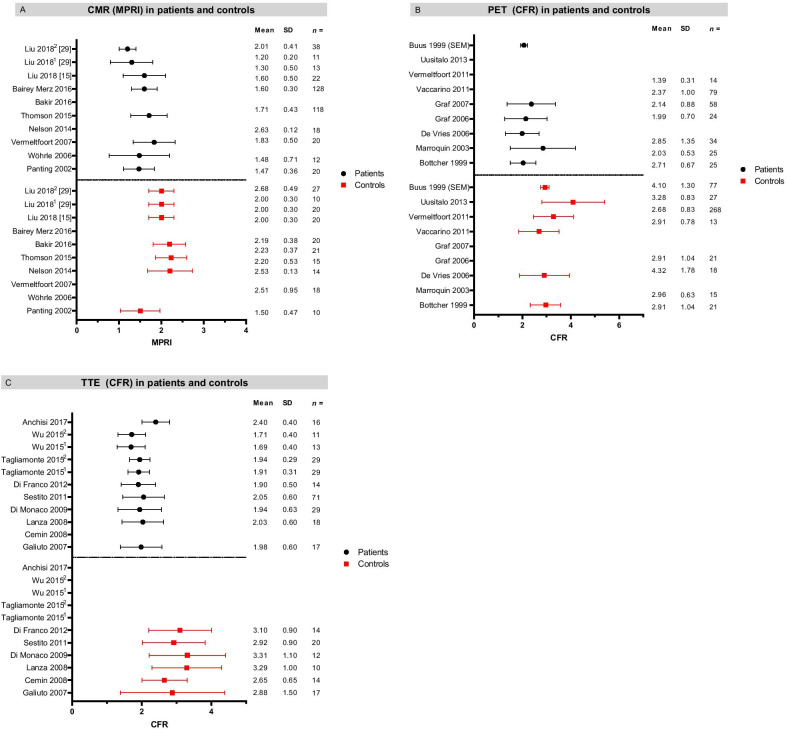
Overview of study outcomes presented as mean ± SD in patients and controls for MPRI by CMR (**a**), CFR by PET (**b**) and CFR by TTE (**c**). For CMR. Error bars are not shown for some studies as some only assessed patient or control subjects. Studies with multiple patient or control groups are indicated by numbers. *CFR* coronary flow reserve, *CMR* cardiac magnetic resonance imaging, *MPRI* myocardial perfusion reserve index, *PET* positron emission tomography, *TTE* transthoracic echocardiography

Unfortunately, the Figure showed all included studies instead of only the studies with the specific measurement mentioned in the Figure caption. The studies that showed a different measure of coronary microvascular dysfunction should have been removed.

The rectified Figure is shown here below, as well as the original article, which has now been updated.
