# Supplementary material for: Evaluation of non-invasive imaging parameters in coronary microvascular disease: a systematic review
Source: BMC Med Imaging. 2021 Jan 6;21:5. doi: 10.1186/s12880-020-00535-7 (PMC7789672; doi:10.1186/s12880-020-00535-7)
Supplement: Supplementary file 1 — Additional file 1. Search Strategy. [file 12880_2020_535_MOESM1_ESM.pdf]

Additional file 1: Search Strategy

PubMed search strategy

(((nonocclusive OR “non occlusive” OR nonobstructive OR “non obstructive” OR “microvascular dysfunction” OR “small vessel disease” OR “cardiac syndrome X” OR “microvascular angina” OR CMD OR MVD)) AND (coronary OR heart OR myocardial OR cardiac)) AND (imaging OR MRI OR CT OR SPECT OR PET OR TTE OR echo)

Embase search strategy

(nonocclusive OR 'non occlusive' OR nonobstructive OR 'non obstructive' OR 'microvascular dysfunction' OR 'small vessel disease' OR 'cardiac syndrome x' OR 'microvascular angina' OR cmd OR mvd) AND (coronary OR heart OR myocardial OR cardiac) AND (imaging OR mri OR ct OR spect OR pet OR tte OR echo)
