# Supplementary material for: Evaluation of non-invasive imaging parameters in coronary microvascular disease: a systematic review
Source: BMC Med Imaging. 2021 Jan 6;21:5. doi: 10.1186/s12880-020-00535-7 (PMC7789672; doi:10.1186/s12880-020-00535-7)
Supplement: Supplementary file 2 — Additional file 2: Table S1. Table S2 Risk of bias assessment according to QUADAS-2. [file 12880_2020_535_MOESM2_ESM.pdf]

Additional file 2: Table S1 Risk of bias assessment according to QUADAS-2.

Author (year)	Risk of bias					Applicability concerns			
	Patient selection	*Index test*	*Reference standard*	*Flow and timing*	*Overall judgement of risk of bias*	*Patient selection*	*Index test*	*Reference standard*	*Overall concern for applicability*
Meeder (1997)	Red	Yellow	Yellow	Yellow	Red	Green	Green	Green	Green
Bottcher (1999)	Red	Yellow	Green	Green	Red	Green	Green	Green	Green
Buus (1999)	Yellow	Yellow	Green	Green	Yellow	Green	Green	Green	Green
Panting (2002)	Yellow	Green	Green	Red	Red	Green	Green	Green	Green
Marroquin (2003)	Red	Yellow	Yellow	Yellow	Red	Green	Green	Green	Green
De Vries (2006)	Green	Yellow	Green	Yellow	Yellow	Green	Green	Green	Green
Graf (2006)	Yellow	Red	Green	Green	Red	Green	Green	Green	Green
Pärkkä (2006)	Yellow	Yellow	Yellow	Green	Yellow	Green	Green	Green	Green
Wöhrle (2006)	Yellow	Green	Green	Green	Yellow	Green	Green	Green	Green
Graf (2007)	Green	Yellow	Green	Yellow	Yellow	Green	Green	Green	Green
Vermeltfoort (2007)	Red	Green	Green	Red	Red	Green	Green	Green	Green
Galiuto (2007)	Yellow	Yellow	Green	Yellow	Yellow	Green	Green	Green	Green
Cemin (2008)	Yellow	Green	Green	Yellow	Yellow	Green	Green	Green	Green
Lanza (2008)	Yellow	Yellow	Green	Green	Yellow	Green	Green	Green	Green
Di Monaco (2009)	Yellow	Yellow	Green	Yellow	Yellow	Green	Green	Green	Green
Mehta (2011)	Green	Yellow	Green	Yellow	Yellow	Green	Green	Green	Green
Scholtens (2011)	Yellow	Yellow	Green	Yellow	Yellow	Green	Green	Green	Green
Sestito (2011)	Green	Yellow	Green	Yellow	Yellow	Green	Green	Green	Green
Vaccarino (2011)	Red	Green	Yellow	Yellow	Red	Green	Green	Green	Green
Vermeltfoort (2011)	Yellow	Yellow	Yellow	Yellow	Yellow	Green	Green	Green	Green
Di Franco (2012)	Green	Green	Green	Yellow	Yellow	Green	Green	Green	Green
Karamitsos (2012)	Yellow	Yellow	Green	Yellow	Yellow	Green	Green	Green	Green
Uusitalo (2013)	Yellow	Yellow	Yellow	Yellow	Yellow	Green	Green	Green	Green
Nelson (2014)	Yellow	Yellow	Green	Yellow	Yellow	Green	Green	Green	Green
Thomson (2015)	Yellow	Green	Green	Yellow	Yellow	Green	Green	Green	Green
Tagliamonte (2015)	Yellow	Yellow	Green	Green	Yellow	Green	Green	Green	Green
Wu (2015)	Yellow	Yellow	Yellow	Yellow	Yellow	Green	Green	Green	Green
Bairey Merz (2016)	Yellow	Yellow	Green	Yellow	Yellow	Green	Green	Green	Green
Bakir (2016)	Red	Yellow	Yellow	Yellow	Red	Green	Green	Green	Green
Mygind (2016)	Green	Yellow	Green	Red	Red	Green	Green	Green	Green
Anchisi (2017)	Red	Yellow	Green	Yellow	Red	Green	Green	Green	Green

Jaarsma (2017)									
Michelsen (2017)									
Liu (2018) (19)									
Liu (2018) (27)									
Zorach (2018)									
Rahman (2019)									

Legenda risk of bias assessment

Low risk of bias or<br>Low concern of applicability	
High risk of bias or<br>High concern of applicability	
Unclear risk of bias or<br>Unclear concern of applicability	
