# Supplementary material for: Evaluation of non-invasive imaging parameters in coronary microvascular disease: a systematic review
Source: BMC Med Imaging. 2021 Jan 6;21:5. doi: 10.1186/s12880-020-00535-7 (PMC7789672; doi:10.1186/s12880-020-00535-7)
Supplement: Supplementary file 3 — Additional file 3: Table S2. Quantification of coronary microvascular dysfunction in CMR studies. [file 12880_2020_535_MOESM3_ESM.pdf]

Additional file 3: Table S2 Quantification of coronary microvascular dysfunction in CMR studies.

Study	Outcome measure	CMR imaging protocol	Patient group n =	Mean (±SD)	Control group n =	Mean (±SD)
Mean ± SD						
Panting (2002)	MPRI	1.5T, adenosine	20	1.47 ± 0.36	10	1.50 ± 0.47
Wöhrle (2006)	MPRI	1.5T, adenosine	12	1.48 ± 0.71	N/A	N/A
Pärkkä (2006)	MPR	1.5T, dipyridamole	N/A	N/A	18	2.51 ± 0.95
Vermeltfoort (2007)	MPRI	1.5T, adenosine	20	1.83 ± 0.50	N/A	N/A
Karamitsos (2012)	CFR (corrected for RPP)	3.0T, adenosine	18	2.63 ± 0.12	14	2.53 ± 0.13
Nelson (2014)	MPRI	1.5T, adenosine	N/A	N/A	15	2.20 ± 0.53
Thomson (2015)	MPRI	1.5T, adenosine	118	1.71 ± 0.43	21	2.23 ± 0.37
Bairey Merz (2016)	MPRI	1.5T, adenosine	128	1.60 ± 0.30	N/A	N/A
Bakir (2016)	MPRI	1.5T, adenosine	N/A	N/A	20	2.19 ± 0.38
Liu (2018) [15]	MPRI	1.5T or 3.0T, adenosine	22	1.60 ± 0.50	20	2.00 ± 0.30
Liu (2018) [29]	MPRI (MPR)	1.5T, adenosine<br>3.0T, adenosine	13<br>11	1.30 ±0.50 (1.50 ±0.60)<br>1.20 ±0.20 (1.8 0±0.70)	20<br>10	2.00 ± 0.30 (2.80 ± 0.50)<br>2.00 ± 0.30 (2.80 ± 0.40)
Rahman (2019)	MPR	3.0T, adenosine	38	2.01 ± 0.41	27	2.68 ±0.49
Median (IQR)						
Jaarsma (2017)	MPR	3.0T, adenosine	12	1.35 (1.09-1.78)	N/A	N/A
Median (range)						
Mehta (2011)	MPRI	1.5T, adenosine	20	1.40 (1.20 – 1.90)	N/A	N/A

Zorach (2018)	MPR	1.5T, regadenoson	46	2.21 (1.95 – 2.69)	20	2.93 (2.76-3.19)
**Abbreviations:** CFR = coronary flow reserve, IQR = interquartile range, MPR = myocardial perfusion reserve, MPRI = myocardial perfusion reserve index, N/A = not available, SD = standard deviation, T = Tesla.						
