# Supplementary material for: Evaluation of non-invasive imaging parameters in coronary microvascular disease: a systematic review
Source: BMC Med Imaging. 2021 Jan 6;21:5. doi: 10.1186/s12880-020-00535-7 (PMC7789672; doi:10.1186/s12880-020-00535-7)
Supplement: Supplementary file 4 — Additional file 4: Table S3. Quantification of coronary microvascular dysfunction in PET studies. Correction for RPP is indicated by + or –. [file 12880_2020_535_MOESM4_ESM.pdf]

## Additional file 4: Table S3 Quantification of coronary microvascular dysfunction in PET studies. Correction for RPP is indicated by + or –.

| Study | Outcome measure | PET imaging protocol | Patient group *n* = | Mean (±SD) | Control group *n* = | Mean (±SD) |
|---|---|---|---|---|---|---|
| *Mean ± SD* | | | | | | |
| Meeder (1997) | MPR (-) | N-13, dipyridamole | 25 | 2.71 ± 0.67 | 21 | 2.91 ± 1.04 |
| Bottcher (1999) | CFR (+) | N-13, dipyridamole | 25 | 2.03 ± 0.53 | 15 | 2.96 ± 0.63 |
| Marroquin (2003) | CFR (-) | N-13, adenosine | 34 | 2.85 ± 1.35 | N/A | N/A |
| Graf (2006) | CFR (+) | N-13, dipyridamole | 58 | 2.14 ± 0.88 | N/A | N/A |
| De Vries (2006) | CFR (+) | N-13, dipyridamole | 24 | 1.99 ± 0.70 | 21 | 2.91 ± 1.04 |
| Pärkkä (2006) | MPR (-) | 15-O labeled water, dipyridamole | N/A | N/A | 18 | 4.32 ± 1.78 |
| Graf (2007) | CFR (-) | N-13, dipyridamole | 79 | 2.37 ± 1.00 | N/A | N/A |
| Scholtens (2011) | MPR (+) | N-13, adenosine | 14 | 1.39 ± 0.31 | 13 | 2.91 ± 0.78 |
| Vaccarino (2011) | CFR (-) | N-13, adenosine | N/A | N/A | 268 | 2.68 ± 0.83 |
| Vermeltfoort (2011) | CFR (+) | 15-O labeled water, adenosine | N/A | N/A | 27 | 3.28 ± 0.83 |
| Uusitalo (2013) | CFR (+) | 15-O labeled water, adenosine or dipyridamole | N/A | N/A | 77 | 4.10 ± 1.30 |
| *Mean ± SEM* | | | | | | |
| Buus (1999) | CFR (-) | N-13, dipyridamole | 16 | 2.06 ± 0.14 | 15 | 2.93 ± 0.17 |
| *Median (IQR)* | | | | | | |

| | | | | | | |
|---|---|---|---|---|---|---|
| Michelsen (2017) | MFR (+) | Rb-82, adenosine | 95 | 2.13 (1.80-2.40) | N/A | N/A |

**Abbreviations:** CFR = coronary flow reserve, IQR = interquartile range, MPR = myocardial perfusion reserve, MFR: myocardial flow reserve, N/A = not available, SD = standard deviation, SEM = standard error of the mean.
