# Supplementary material for: Evaluation of non-invasive imaging parameters in coronary microvascular disease: a systematic review
Source: BMC Med Imaging. 2021 Jan 6;21:5. doi: 10.1186/s12880-020-00535-7 (PMC7789672; doi:10.1186/s12880-020-00535-7)
Supplement: Supplementary file 5 — Additional file 5: Table S4. Quantification of coronary microvascular dysfunction in TTE studies. [file 12880_2020_535_MOESM5_ESM.pdf]

Additional file 5: Table S4 Quantification of coronary microvascular dysfunction in TTE studies.

Study	Outcome measure	TTE imaging protocol	Patient group *n* =	Mean (±SD)	Control group *n* =	Mean (±SD)
Mean ± SD						
Galiuto (2007)	CFR	PW Doppler, distal LAD, adenosine	17	1.98 ± 0.60	17	2.88 ± 1.50
Cemin (2008)	CFR	PW Doppler, distal LAD, adenosine	N/A	N/A	14	2.65 ± 0.65
Lanza (2008)	CFR	Doppler ST, mid-distal LAD, adenosine	18	2.03 ± 0.60	10	3.29 ± 1.00
Di Monaco (2009)	CFR	Doppler ST mid-distal LAD, adenosine	29	1.94 ± 0.63	12	3.31 ± 1.10
Sestito (2011)	CBF	Doppler ST, mid-distal LAD, adenosine	71	2.05 ± 0.60	20	2.92 ± 0.90
Di Franco (2012)	CBF	Doppler ST, mid-distal LAD, adenosine	14	1.90 ± 0.50	14	3.10 ± 0.90
Tagliamonte (2015)	CFR	Doppler ST, mid-distal LAD, dipyridamole	29 (ranolazine)<br>29 (placebo)	1.91 ± 0.31<br>1.94 ± 0.29	N/A	N/A
Wu (2015)	CBFV	PW Doppler, mid-distal LAD, nitroglycerin	13 (placebo)<br>11 (ISMN)	1.69 ± 0.40<br>1.71 ± 0.40	N/A	N/A
Anchisi (2017)	CFR	Color Doppler, mid-distal LAD, dipyridamole	16	2.40 ± 0.40	N/A	N/A
Median (IQR)						
Mygind (2016)	CFVR	PW Doppler,	963	2.33 (1.98–2.76)	N/A	N/A

		LAD, dipyridamole or MCE				
Michelsen (2017)	CFVR	PW Doppler, LAD, dipyridamole or MCE	102	1.99 (1.64–2.43)	N/A	N/A

Abbreviations: CFR = coronary flow reserve, CBF = coronary blood flow, CBFV = coronary blood flow velocity, CFVR = coronary flow velocity reserve, PW = pulsed-waved, ST = spectral tracing, MCE = myocardial contrast echocardiography, LAD = left anterior descending artery, IQR = interquartile range, N/A = not available, SD = standard deviation.
