# Supplementary material for: Evaluation of non-invasive imaging parameters in coronary microvascular disease: a systematic review
Source: BMC Med Imaging. 2021 Jan 6;21:5. doi: 10.1186/s12880-020-00535-7 (PMC7789672; doi:10.1186/s12880-020-00535-7)
Supplement: Supplementary file 6 — Additional file 6: Table S5. Definition of nonobstructive CAD in included studies. [file 12880_2020_535_MOESM6_ESM.pdf]

Additional file 6: Table S5 Definition of nonobstructive CAD in included studies.

Author (year)	Definition nonobstructive CAD
Meeder (1997)	Completely normal coronary arteries without any vessel irregularities on CAG.
Bottcher (1999)	Completely normal coronary arteriography.
Buus (1999)	Normal CAG with smooth coronary arteries.
Panting (2002)	Completely normal results on coronary angiography, with no inducible spasm on ergonovine-provocation testing.
Marroquin (2003)	Normal or minimal luminal irregularities on CAG (<50% stenosis)
De Vries (2006)	Normal coronary findings on angiography.
Graf (2006)	Normal angiogram.
Pärkkä (2006)	N/A
Wöhrle (2006)	Smooth vessel contours without lumen irregularities on CAG.
Galiuto (2007)	Angiographically normal coronary arteries.
Graf (2007)	Normal coronary angiogram.
Vermeltfoort (2007)	Completely normal results from coronary angiography.
Cemin (2008)	N/A
Lanza (2008)	Angiographically normal epicardial coronary arteries.
Di Monaco (2009)	Totally normal coronary arteries at angiography.
Mehta (2011)	<50% epicardial coronary stenosis in all epicardial coronary arteries on CAG.
Scholtens (2011)	Normal coronary angiogram.
Sestito (2011)	Smooth coronary arteries at angiography. Coronary artery spasm had been excluded according to intracoronary or systemic ergonovine test in patients who reported angina episodes also at rest
Vaccarino (2011)	N/A
Vermeltfoort (2011)	N/A
Di Franco (2012)	Angiographically normal coronary arteries.
Karamitsos (2012)	Angiographically smooth normal epicardial coronary arteries on CAG.
Uusitalo (2013)	N/A
Nelson (2014)	N/A
Thomson (2015)	Exclusion: >50% luminal diameter stenosis in 1 epicardial coronary artery.
Tagliamonte (2015)	<70% coronary stenosis in all epicardial coronary arteries on CAG.
Wu (2015)	Normal coronary angiography.
Bairey Merz (2016)	<50% epicardial coronary stenosis in all epicardial coronary arteries.
Bakir (2016)	N/A
Mygind (2016)	<50 % stenosis of epicardial vessels assessed by diagnostic invasive CAG.
Anchisi (2017)	Normal coronary angiography.
Jaarsma (2017)	Normal coronary arteries or minimal vessel wall irregularities (<25% stenosis) on CAG.
Michelsen (2017)	<50% stenosis on CAG.

Liu (2018) (19)	Angiographic NOCAD (<50% visual stenosis) on CAG.
Liu (2018) (27)	FFR ≥0.8 and IMR ≥25 U measured with invasive CAG.
Zorach (2018)	Exclusion: >50% stenosis in at least one coronary artery or FFR <0.8 in at least one coronary artery on CAG.
Rahman (2019)	CAG result of <30% diameter stenosis and/or fractional flow reserve >0.80 and invasive CFR < 2.5
